# XPC is an RNA polymerase II cofactor recruiting ATAC to promoters by interacting with E2F1

**DOI:** 10.1038/s41467-018-05010-0

**Published:** 2018-07-04

**Authors:** B. Bidon, I. Iltis, M. Semer, Z. Nagy, A. Larnicol, A. Cribier, M. Benkirane, F. Coin, J-M. Egly, N. Le May

**Affiliations:** 1 Institut de Génétique et de Biologie Moléculaire et Cellulaire, BP 163 Illkirch Cedex, 67404 C.U. Strasbourg, France; 2 Centre National de la Recherche Scientifique, UMR7104, 67404 Illkirch, France; 3 Institut National de la Santé et de la Recherche Médicale, U1258, 67404 Illkirch, France; 4 Université de Strasbourg, 67404 Illkirch, France; 5 Institut de Génétique Humaine, Laboratoire de Virologie Moléculaire, Université de Montpellier, CNRS UMR9002, 34000 Montpellier, France

## Abstract

The DNA damage sensor XPC is involved in nucleotide excision repair. Here we show that in the absence of damage, XPC co-localizes with RNA polymerase II (Pol II) and active post-translational histone modifications marks on a subset of class II promoters in human fibroblasts. XPC depletion triggers specific gene down-expression due to a drop in the deposition of histone H3K9 acetylation mark and pre-initiation complex formation. XPC interacts with the histone acetyltransferase KAT2A and specifically triggers the recruitment of the KAT2A-containing ATAC complex to the promoters of down-expressed genes. We show that a strong E2F1 signature characterizes the XPC/KAT2A-bound promoters and that XPC interacts with E2F1 and promotes its binding to its DNA element. Our data reveal that the DNA repair factor XPC is also an RNA polymerase II cofactor recruiting the ATAC coactivator complex to promoters by interacting with the DNA binding transcription factor E2F1.

## Abstract (web summary)

XPC plays an important role in the nuclear exicision repair pathways. Here the authors show that in addition, XPC plays a role in transcription regulation by interacting with KAT2A and E2F1 and recruiting the ATAC coactivator complex to promoters.

## Introduction

Gene expression is constantly compromised by genotoxic stress that challenges genome integrity and requires the function of several DNA repair pathways to remove DNA lesions. This implies that there must be connections between the disparate events of transcription and DNA repair to orchestrate the expression and repair of genes. A link between DNA repair and transcription was first established after the discovery of a nucleotide excision repair (NER) sub-pathway removing DNA lesions located on the actively transcribed strand blocking elongating RNA polymerase II (Pol II) called the transcription coupled repair (TCR)^1^. This was followed by the characterization of the basal transcription TFIIH as a NER factor involved both in TCR and in global genome repair (GGR), eliminating DNA damage from the entire genome^2,3^. This interplay is even tighter, since studies also revealed roles for the other NER factors (CSB, XPC, XPA, XPG, and XPF/ERCC1) in gene expression^4–6^.

Understanding the roles played by NER factors is of prime importance not only to unveil the molecular details of gene expression but also to understand how mutations in their corresponding genes give rise to several human autosomal recessive disorders like Xeroderma pigmentosum (XP), Cockayne syndrome (CS), and trichothiodystrophy (TTD). Patients bearing mutations in *XPC* only develop XP (XP-C) and represent the most frequent NER-defective group. XP is clinically characterized by an extreme sensitivity to ultraviolet (UV) rays from sunlight. XP patients develop severe sunburns and are highly susceptible to develop tumors on sunlight-exposed areas of the skin, including melanoma and squamous cell carcinoma^7^. XP individuals also present increased susceptibility for lung, breast, and colorectal cancers with possible neurological issues^8^. The XP pathology has been primarily defined as a DNA repair syndrome due to the inability of patients’ cells to eliminate DNA lesions. However, studies during the last decade suggest that some of their phenotypes may also stem from transcriptional deregulations^9^.

Upon NER, XPC, with its partner hHR23B, recognizes all along the genome DNA-distorting lesions inflicted by endogenous and exogenous genotoxic attacks like UV irradiation, thereby initiating only the GGR sub-pathway^10^. Several observations suggest that XPC is additionally involved in the modifications of the chromatin environment surrounding the DNA lesions, including histone post-translational modifications (PTMs)^11–13^. We have shown earlier that NER factors are associated with the Pol II transcription machinery and are sequentially recruited at the promoter of transcribed genes^6^. The presence of these NER factors at promoter is required to achieve optimal chromatin remodeling, including histone PTMs as well as active DNA demethylation, DNA break induction, and gene looping^6,14^. Furthermore, a complex containing XPC and Oct4/Sox2 has been identified as a coactivator in embryonic stem (ES) and induced pluripotent stem cells^15,16^. Although the involvement of XPC in transcription is established, its mechanistic role remains largely elusive as well as its transcriptional partners in the pre-initiation complex.

In the present study, we investigated the roles of XPC in class II gene expression. We first assessed the genome-wide localization of XPC and revealed that in the absence of a genomic stress, XPC is mainly recruited to the promoters of active genes where it co-localizes with Pol II. Depletion of XPC leads to deregulation of Pol II recruitment and altered histone marks at promoters, including H3K9ac. We further identified an interaction between XPC and the histone acetyltransferase (HAT) lysine acetyltransferase 2A KAT2A (or GCN5). Our data indicated that XPC, through its interaction with KAT2A, could be associated with both ATAC and SAGA complexes but that only ATAC is detected at the promoters of XPC-dependent genes. GREAT analysis unveiled that a strong E2F1 signature characterizes genes regulated by XPC/KAT2A. We showed that E2F1 interacts with XPC and is required for its localization at the promoter of activated genes. We subsequently identified a complex, including XPC, E2F1, and KAT2A that is specifically recruited to XPC-dependent promoters, conditioning the presence of the ATAC complex and the deposition of the H3K9ac mark. Altogether, our results establish XPC as a cofactor of Pol II, interacting with a transcription factor and recruiting a coactivator complex to remodel chromatin and initiate class II gene expression.

## Results

### Enrichment of XPC on promoters in the absence of genomic stress

**Fig. 1:**
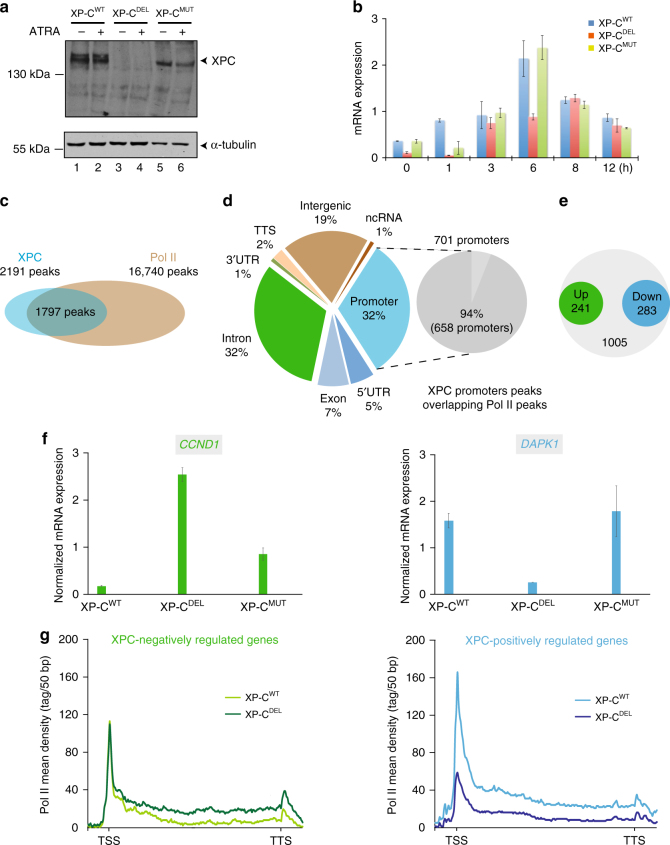
Co-localization of XPC and Pol II genome-wide. **a** Relative protein expression of XPC and α-tubulin analyzed by western blotting of whole-cell extract from XP-C^WT^, XP-C^DEL^, and XP-C^MUT^ fibroblasts, in presence or absence of ATRA. **b** Relative mRNA expression of *RARβ2* in XP-C^WT^, XP-C^DEL^, and XP-C^MUT^ fibroblasts, after ATRA treatment in 12-h time-course experiment. Error bars represent the standard deviation of three independent experiments. **c** Overlapping of MACS14-determined peaks for both Pol II and XPC in XP-C^WT^ cells after ATRA induction. XPC and Pol II peaks correspond to recurrent peaks found in three independent ChIP-seq experiments. **d** HOMER annotation of the 2191 XPC peaks and proportion of promoter annotated peaks to overlap with a Pol II peak. **e** Expression level of XPC-bound genes identified by ChIP-seq were determined by RNA-seq by comparing both ATRA-treated XP-C^WT^ and XP-C^DEL^ cells. Differential expression analysis of these genes in XP-C^WT^, compared to XP-C^DEL^, was then computed using EdgeR represented as a circle plot. XPC-negatively regulated genes (upregulated in XP-C^DEL^) are enlightened in green and XPC-positively regulated genes (downregulated in XP-C^DEL^) in blue. **f** Relative mRNA expression of two genes, *CCND1* as XPC-negatively regulated one and *DAPK1* as XPC-positively regulated one, after ATRA treatment in XP-C^WT^, XP-C^DEL^, and XP-C^MUT^ cells. Error bars represent the standard deviation of three independent experiments. **g** Diagrams representing the mean tag density of Pol II ChIP-seq experiment for XPC-negatively regulated and -positively regulated genes, as they were previously determined in XP-C^WT^ and XP-C^DEL^ cells

To investigate the involvement of XPC upon transcription, we set forward to analyze transcription genome-wide in fibroblasts defective in endogenous XPC, hereafter called XP-C^DEL^ cell line (Fig. 1a)^17,18^. Wild-type GFP-XPC cDNA was re-introduced into XP-C^DEL^ cells to be used as positive control cell line, hereafter called XP-C^WT^^19^. All along the study, we also compared these two cell lines to another XP-C patient cell line bearing the XPC p.Pro334His mutation with mild phenotypes, in which XPC is mutated and expressed (Fig. 1a) and hereafter called XP-C^MUT^^17^.

By employing the all-*trans* retinoic acid (ATRA) stimulus, which induces a well-characterized modification of the cellular transcriptional program^6,14^, we sought to identify the XPC genomic distribution and its impact on gene expression in a well-defined system. We first analyzed the RA response in the three cell lines by studying the transactivation of *RARß2* upon ATRA treatment. We observed a correlation at 6 h post ATRA treatment between *RARß2* mRNA induction and recruitment of the transcriptional machinery (including RAR, Pol II, and TFIIH kinase CDK7), together with the NER factors (XPC, XPA, and XPG) at the *RARß2* promoter in both XP-C^WT^ and XP-C^MUT^ cells (Fig. 1b and Supplementary Figure 1A). However, *RARß2* transactivation and the concomitant recruitment pattern were largely abolished in XP-C^DEL^ cells characterized by the absence of XPC (Fig. 1b and Supplementary Figure 1A). We thus performed chromatin immunoprecipitation (ChIP) followed by high-throughput DNA sequencing (ChIP-seq) in both XP-C^WT^ and XPC^DEL^ cells that were treated during 6 h with ATRA using antibodies directed against green fluorescent protein (GFP) and Pol II. Three independent series using XP-C^WT^ cells yielded 2191 and 16, 440 peaks for GFP-tagged XPC and Pol II, respectively. We identified 1797 common binding events representing 82% of the XPC peaks (Fig. 1c). Using HOMER annotation, we observed that 32% of the XPC-binding events appeared at promoter regions (Fig. 1d). Moreover, the combination of 5′ untranslated region and exon-annotated peaks (mostly located in the very first one) represented 44% of the XPC peaks, all located near the transcription starting site (TSS). Interestingly, 94% of these XPC-bound promoters also harbored an enrichment of Pol II (Fig. 1d).

RNA-seq analysis has been performed in parallel to ChIP-seq in the ATRA-treated XP-C^DEL^ cells and compared to XP-C^WT^. Peak annotation of the 1797 common binding events between XPC and Pol II identified 1529 genes, whose expression varied after ATRA treatment indicating that they were presumably regulated by the presence of XPC. We observed that 283 genes were downregulated in the absence of XPC, whereas 241 genes were upregulated (Fig. 1e and Supplementary Data 1). Based on these results, we defined two groups of genes: one containing 283 genes positively regulated by XPC and the other containing 241 genes negatively regulated by XPC. The top-ranking Gene Ontology (GO) biological process with significant *p* values included chromatin-structure-related terms such as chromatin/nucleosome assembly, DNA packaging, and protein-DNA organization that is in agreement with the well-defined role of XPC in genomic stability (Supplementary Figure 1B). As expected, the top-ranking GO biological pathways were related to RAR-targeted genes but we also noticed numerous interferon (IFN) gamma-related genes (Supplementary Data 2).

We subsequently validated RNA-seq by reverse transcription-PCR in XP-C^WT^ and XP-C^DEL^ cells, using *CCND1* as representative of XPC-negatively regulated genes and *DAPK1*, *HOXB13*, and *LRRC11* as representatives of XPC-positively regulated genes and observed that the expression of these genes was not modified by the XPC p.Pro334His mutation found in XP-C^MUT^ cells (Fig. 1f and Supplementary Figure 1E).

We next determined the mean tag density of Pol II for both XPC-positively and -negatively regulated genes in XP-C^DEL^ cells. We observed a lower occupancy of Pol II at TSS and along the gene body of the XPC-positively regulated genes in the absence of XPC (Fig. 1g, right panel), while Pol II enrichment was higher mainly along the gene body for the XPC-negatively regulated genes (Fig. 1g, left panel). ChIP experiments targeting the *CCND1* or *DAPK1* promoter recapitulated ChIP-seq data (see also additional XPC-positively regulated genes in Supplementary Figure 1F, top panels). Finally, to rule out the possibility that our observations were due to an overexpression of XPC in the rescued XP-C^WT^ cells, we tested and compared rescued XP-C^WT^ fibroblasts with MRC-5 cells showing similar inductions of XPC-target genes and comparable recruitments by ChIP upon ATRA treatment in these two cell lines (Supplementary Figure 1C-D).

Collectively, our findings established an enrichment of XPC around promoters that strongly correlates with Pol II at TSSs, regulating more than 500 genes.

### Enrichment of XPC correlates with positive histone PTMs

**Fig. 2:**
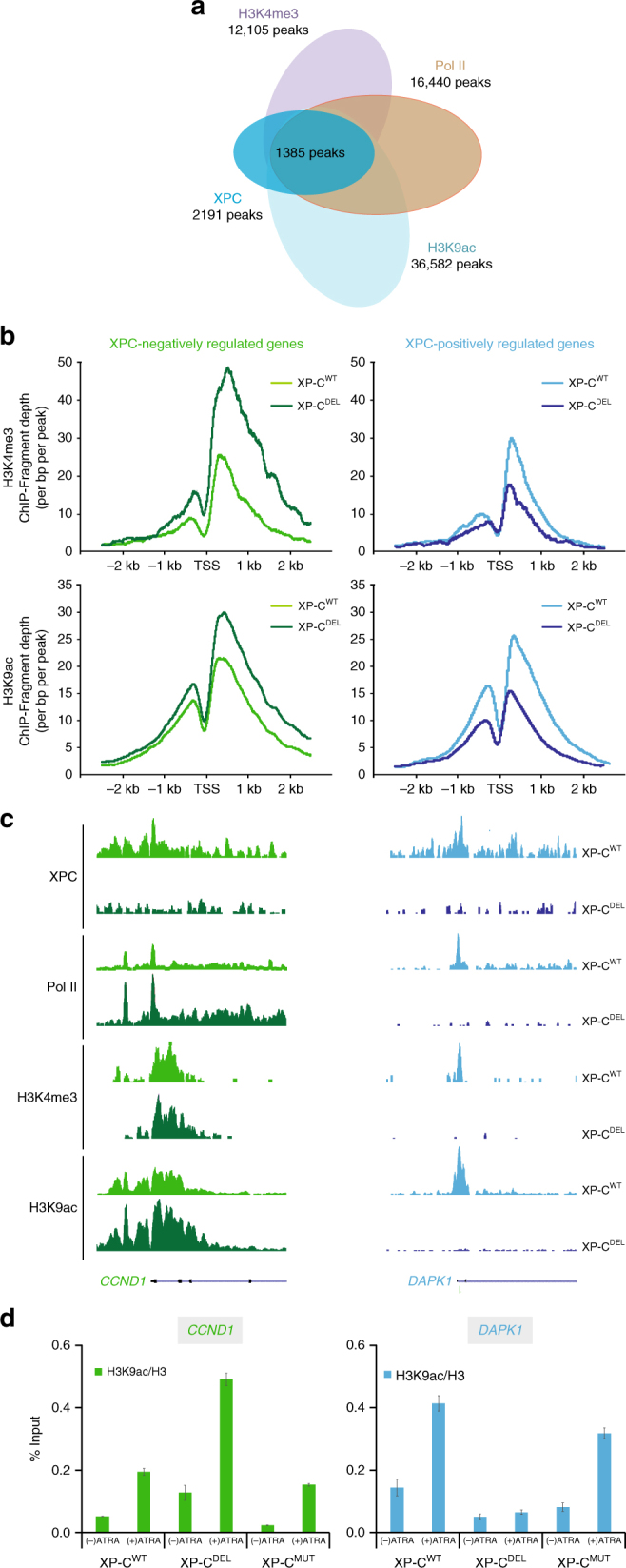
Association of XPC-bound promoters with active histone PTMs. **a** Overlapping of peaks for Pol II and XPC with PTMs H3K4me3 and H3K9ac in XP-C^WT^ cells after ATRA induction. These peaks correspond to recurrent peaks found in three independent ChIP-seq experiments. **b** Diagrams representing the fragment depth of H3K9ac and H3K4me3 ChIP-seq experiment for XPC-negatively regulated (left panels) and XPC-positively regulated (right panels) genes, as they were previously determined in XP-C^WT^ and XP-C^DEL^ cells. **c** UCSC genome browser for XPC, Pol II, H3K4me3, and H3K9ac at *CCND1* and *DAPK1* promoters as XPC-negatively regulated and XPC-positively regulated genes, respectively. **d** Relative enrichment of H3K9ac/H3 monitored by ChIP at *CCND1* and *DAPK1* promoters as XPC-negatively regulated and XPC-positively regulated genes. Error bars represent the standard deviation of three independent experiments

We next investigated the correlation between the presence of XPC and the deposition of positive histone marks at TSS. We performed a comparative ChIP-seq analysis using both ATRA-treated XP-C^DEL^ and XP-C^WT^ cells for H3K9ac and H3K4me3 marks. Among the 1797 common binding events between XPC and Pol II detected in XP-C^WT^ cells, 1756 (98%) were localizing with H3K9ac and 1385 (78%) with both H3K9ac and H3K4me3 (Fig. 2a). Interestingly, ChIP-seq data comparison between XP-C^WT^ and XP-C^DEL^ cells, considering previously identified XPC-regulated genes, showed a correlation between their expression levels, the enrichment of Pol II, and the deposition of the transcription-positive histone marks. Indeed, the fragment depth for H3K9ac and H3K4me3 was higher at promoters of the XPC-negatively regulated genes while they remained lower for XPC-positively regulated genes in XP-C^DEL^ cells compared to XP-C^WT^ cells (Fig. 2b). Distribution of these histones marks along the representative genes *CCND1* and *DAPK1* is shown in Fig. 2c and ChIP on their promoters in Fig. 2d (see also supplemental genes in Supplementary Figure 1F, lower panels). In contrast, and in agreement with the normal expression of *CCND1* and *DAPK1* in XP-C^MUT^ cells compared to XP-C^WT^ cells (Fig. 1f), the deposition of H3K9ac and H3K4me3 on the promoter of these genes was not modified in XP-C^MUT^ cells (Fig. 2d and Supplementary Figure 1F, lower panels).

To exclude the false positive results due to the use of XPC^WT^ cells overexpressing XPC, we also analyzed HeLa cells constitutively expressing short hairpin RNA either directed against XPC (shXPC), XPA (shXPA), or scrambled (shCtrl) overtime treated with ATRA (Supplementary Figure 2A, left panel). We observed lower induction of mRNA *RARß2* synthesis, 6 h post ATRA treatment in shXPC and shXPA cells, compared with shCTL cells (Supplementary Figure 2A, middle panel). We also determined the transcriptome profiles of shCtrl, shXPC, and XP-C^DEL^ cells by comparing mRNA induction in untreated and 6 h ATRA-treated cells. In shCtrl cells, 77 genes show a fold induction >2, including several well-characterized genes, such as *RARß2* (Supplementary Figure 2B, Supplementary Data 4, and Supplementary Methods). Surprisingly, ~90% of genes activated in shCtrl showed a lower induction in both shXPC and XP-C^DEL^ cells (Supplementary Figure 2B and Supplementary Data 4). The *RARß2* mRNA induction at 6 h post ATRA treatment in shCtrl cells followed the recruitment of the transcriptional machinery together with the NER factors at the promoter (Supplementary Figure 2A, right panels)^6^. This concomitant recruitment pattern was abolished in shXPC and shXPA cells, although XPC was still detected in the latter one (Supplementary Figure 2A, right panels). While the deposition of H3K9ac and H3K4me3 was detected after ATRA treatment at the promoter upon *RARß2* transactivation in shCtrl cells, such enrichment was not observed in shXPC cells. Similar results concerning the deposition of histone marks at *RARß2* promoter were obtained in the corresponding XP-C^WT^ and XP-C^DEL^ cells (Supplementary Figure 2D, right panel). Interestingly, the deposition of the two histone marks was not disturbed by the absence of XPA in shXPA cells in which normal recruitment of XPC was detected (Supplementary Figure 2D, left panel).

Altogether, these results indicated that the genome-wide recruitment of XPC at promoters correlated with transcriptionally active H3K9ac and H3K4me3 marks. They also indicated that XPC was recruited to promoters before XPA in order to initiate histone PTMs at the TSS.

### XPC and KAT2A are recruited to XPC-positively regulated genes

We next wondered which enzyme(s) could be responsible for the histone marks deposition at the TSS of XPC-regulated genes. Given the role of KAT2A in the deposition of H3K9ac in transcription initiation^20^, we assessed whether the variations of H3K9ac at promoters targeted by XPC could be related to this HAT.

**Fig. 3:**
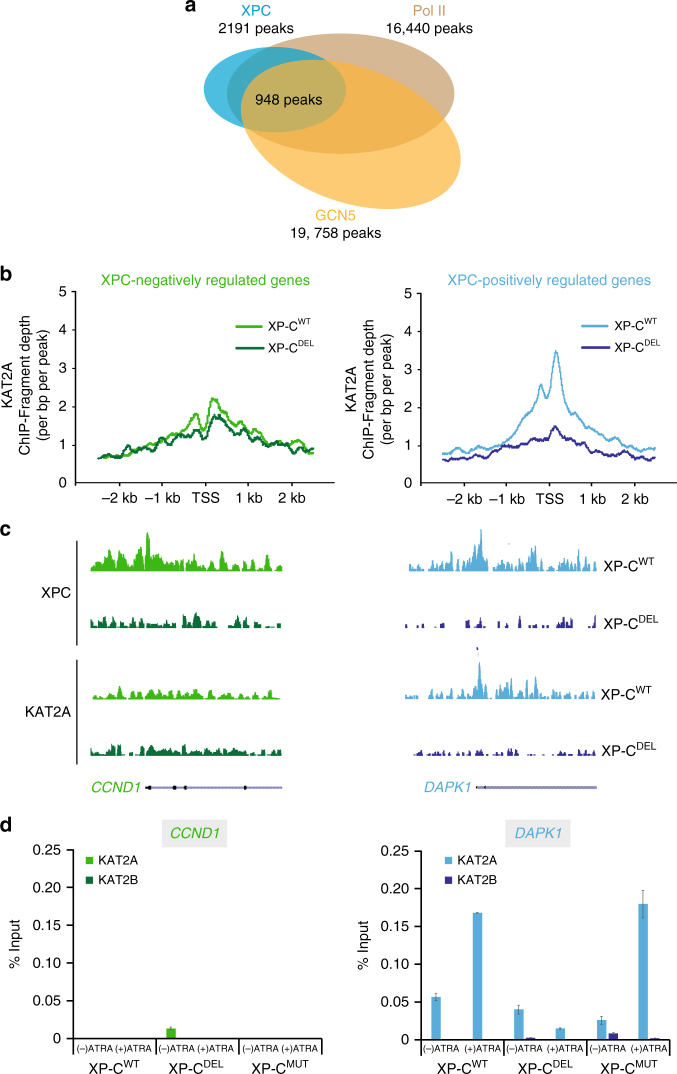
Association of XPC-bound promoters with KAT2A. **a** Overlapping of peaks for Pol II and XPC with KAT2A peaks in XP-C^WT^ cells after ATRA induction. These peaks correspond to recurrent peaks found in three independent ChIP-seq experiments. **b** Diagrams representing the fragment depth of KAT2A ChIP-seq experiment for XPC-negatively regulated (left panels) and XPC-positively regulated (right panels) genes, as they were previously determined in XP-C^WT^ and XP-C^DEL^ cells. **c** UCSC genome browser for XPC and KAT2A at *CCND1* and *DAPK1* promoters as XPC-negatively regulated and XPC-positively regulated genes, respectively. **d** Occupancy of KAT2B and KAT2A monitored by ChIP at *CCND1* and *DAPK1* promoters as XPC-negatively regulated and XPC-positively regulated genes. Error bars represent the standard deviation of three independent experiments

We thus performed ChIP-seq in both XP-C^WT^ and XP-C^DEL^ cells treated 6 h with ATRA, using antibodies directed against KAT2A, and obtained 19, 758 genomic locations for KAT2A compared with input. Using HOMER annotation, we observed almost 50% of these KAT2A-binding events appearing at promoter regions (Supplementary Figure 3A). Among the previously determined XPC and Pol II co-occupied positions, we identified 948 common binding events with KAT2A (Fig. 3a). Interestingly, KAT2A was absent from promoters of the XPC-positively regulated genes in XP-C^DEL^ cells lacking XPC, which correlates with a decrease in H3K9ac (Fig. 3b, right panel compared with Fig. 2b). Surprisingly, KAT2A was detected around TSS of XPC-positively regulated genes only in the presence of XPC in XP-C^WT^ cells while the HAT was hardly detected at XPC-negatively regulated genes with or without XPC, in XP-C^WT^, or XP-C^DEL^, respectively (Fig. 3b). Distribution of KAT2A along the representative genes *CCND1* and *DAPK1* is shown in Fig. 3c. We confirmed by ChIP that KAT2A recruitment correlated with an increase H3K9ac at the promoter of *DAPK1* in XP-C^WT^ cells upon ATRA treatment while the HAT was not detected at *CCND1* (Fig. 3d compared with Fig. 2d and also Supplementary Figure 3B for two additional genes). This recruitment was lost for *DAPK1* in the absence of XPC in XP-C^DEL^ cells concomitantly with the decreased histone PTMs, while we did not observe any change in XP-C^MUT^ cells (Fig. 3d compared with 2D). Interestingly, we did not detect the closely related paralog of KAT2A, KAT2B (or PCAF)^20,21^, on the representative *CCND1* and *DAPK1* promoters by ChIP (Fig. 3d and Supplementary Figure 3B for additional genes). We also detected KAT2A concomitantly with an increase of H3K9ac signal at the activated *RARß2* promoter in shCTL, XP-C^MUT^, XP-C^WT^, and shXPA cells (Supplementary Figure 2D). Interestingly, KAT2A was not detected in the absence of XPC in XP-C^DEL^ and shXPC cells (Supplementary Figure 2D). Finally, ChIP-reChIP assays showed that XPC and KAT2A co-occupied *RARß2* promoter in shCtrl but not in shXPC cells, using both combinations of co-immunoprecipitation (co-IP; XPC/KAT2A or KAT2A/XPC) (Supplementary Figure 3C). These results are comparable to the XPC/Pol II co-occupancy detected at this promoter (Supplementary Figure 3C)^6^. Note that KAT2A was stable in all the cell lines (Supplementary Figure 2C). Taken together, our results point to a correlation between the recruitment of XPC and KAT2A and the deposition of H3K9ac at promoters of XPC-positively regulated genes.

### XPC recruits ATAC to XPC-positively regulated genes

**Fig. 4:**
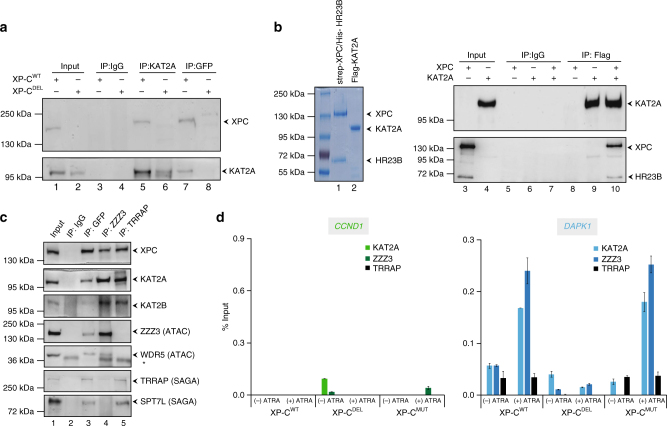
XPC interacts directly with KAT2A. **a** Immunoprecipitation performed on nuclear extract of untreated XP-C^WT^ or XP-C^DEL^ fibroblasts with antibody against GFP, KAT2A, or IgG. **b** Blue staining of recombinant Flag-KAT2A and duplex strep-XPC/His-HR23B (left panel). In vitro co-immunoprecipitation assay performed by antibodies against Flag tag and IgG with the recombinant protein Flag-KAT2A and the duplex strep-XPC/His-HR23B. **c** Immunoprecipitation performed on nuclear extract of untreated XP-C^WT^ fibroblasts with antibody against TRRAP, ZZZ3, GFP, or IgG. Western blot was revealed with antibodies directed against GFP, KAT2A, KAT2B, ZZZ3, WDR5 (ATAC), TRRAP, and SPT7L (SAGA). **d** ChIP experiment monitoring the recruitment of KAT2B, KAT2A, ZZZ3, and TRRAP at *CCND1* and *DAPK1* promoters, before and after ATRA treatment. Error bars represent the standard deviation of three independent experiments

To deepen the correlation between XPC and KAT2A established above, we next assessed their co-location in a complex and their putative interaction. KAT2A co-precipitated a complex containing XPC in nuclear extracts from untreated XP-C^WT^ (Fig. 4a). As expected, such co-precipitation was not detected using nuclear extracts from untreated XP-C^DEL^. Furthermore, recombinant KAT2A was able to pull down the recombinant tagged heterodimer XPC/hHR23B in vitro indicating a direct interaction between them (Fig. 4b).

KAT2A is found in two functionally distinct coactivator complexes, SAGA (Spt Ada Gcn5 Acetyltransferase) and ATAC (Ada Two A Containing)^20,22^. These complexes contain either KAT2A or KAT2B. We thus investigated whether the complex containing XPC and KAT2A could be associated with SAGA and/or ATAC. IP experiments using nuclear extracts from untreated XP-C^WT^ showed that both SAGA and ATAC subunits (TRRAP and ZZZ3, respectively) co-precipitated the complex containing either XPC or KAT2A (Supplementary Figure 4A). IPs of SAGA or ATAC performed with antibodies against the specific subunits TRRAP and ZZZ3, respectively, led to the detection of XPC (Fig. 4c). Interestingly, KAT2B was not immunoprecipitated by XPC although it was detected both in the SAGA and ATAC complexes, indicating the presence of a complex containing XPC and KAT2A HAT in untreated XP-C^WT^ cells (Fig. 4c). We noticed that the integrity of the ATAC and SAGA complexes was not altered by the absence of XPC in XP-C^DEL^ cells (Supplementary Figure 4A).

To determine which complex was recruited with XPC at promoters of XPC-dependent genes, we performed ChIP analysis on *CCND1* and *DAPK1*. As expected, neither ZZZ3 (ATAC subunit) nor TRRAP (SAGA subunit) was detected at the promoter of the XPC-negatively regulated *CCND1* gene in XP-C^DEL^, XP-C^MUT^, or XP-C^WT^ cells in which KAT2A was not recruited (Fig. 4d, left panel). Interestingly, we only observed enrichment of ZZZ3 and KAT2A at the *DAPK1* promoter upon ATRA treatment in XP-C^WT^ and XP-C^MUT^ cells, while neither TRRAP nor KAT2B was detected (Figs. 4d, right panel, and 3d, also Supplementary Figure 4B for supplemental genes). The detection of ZZZ3 at the *DAPK1* promoter was abolished in XP-C^DEL^ cells (Fig. 4d, also Supplementary Figure 4B for supplemental genes). In contrast, TRRAP and ZZZ3 were detected at SAGA-targeted *RBBP5* and ATAC-targeted *SNC16* in XP-C^WT^ and XP-C^DEL^ cells, respectively (Supplementary Figure 4C)^23^.

We also analyzed by ChIP the SAGA vs. ATAC complex recruitment at the *RARß2* promoter in shCtrl, shXPC, and shXPA cells. While only ZZZ3 and KAT2A were readily recruited, no TRRAP could be observed at this promoter upon ATRA treatment in shCtrl or shXPA cells. ZZZ3 and KAT2A accumulation was lost in shXPC cells, indicating that the recruitment of the ATAC complex required the presence of XPC (Supplementary Figure 4D).

Altogether, our findings indicated that XPC not only interacts with both ATAC and SAGA but also plays an active role in transcription via the specific recruitment of the ATAC complex to promoters upon transactivation.

### XPC is recruited to E2F1-dependent genes after ATRA treatment

**Fig. 5:**
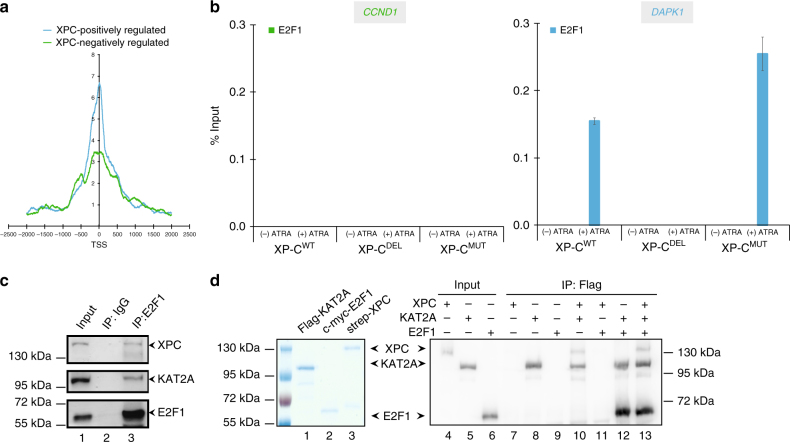
Ménage-à-trois between XPC, KAT2A, and E2F1. **a** Diagrams representing the fragment depth of E2F1 from ENCODE ChIP-seq data for XPC-negatively regulated (green) and XPC-positively regulated (blue) genes, as they were previously determined in XP-C^WT^ and XP-C^DEL^. **b** ChIP experiment investigating the occupancy of E2F1 at *CCND1* and *DAPK1* promoters, with or without ATRA treatment. Error bars represent the standard deviation of three independent experiments.**c** Immunoprecipitation performed on nuclear extracts from untreated XP-C^WT^ fibroblasts with antibody against E2F1 or IgG. Western blot was revealed with antibodies directed against E2F1, XPC, and KAT2A. **d** Left panel; blue staining of recombinant Flag-KAT2A, duplex Strep-XPC/His-HR23B, and c-myc-E2F1. Right panel; in vitro co-immunoprecipitation assay performed by antibodies against Flag tag with the recombinant protein Flag-KAT2A, the duplex Strep-XPC/His-HR23B, and c-myc-E2F1

In the above results, we established that XPC is present in the ATAC complex via KAT2A, triggering active histone PTMs at the promoter of a subset of genes. We then subsequently searched for specific DNA motifs associated with XPC/ATAC-bound promoters that could explain the recruitment of XPC to these promoters. A GREAT analysis indicated enrichment of E2F1 transcription factor-binding motif at 89% of the 283 promoters of XPC-positively regulated genes, including *DAPK1* and *RARß2* (Supplementary Figure 5A). Interestingly, the motif was rarely detected at XPC-negatively regulated genes. We next performed a meta-analysis using E2F1 ChIP-seq data from the ENCODE project by selecting only XPC-positively or -negatively regulated genes, and observed a stronger E2F1 enrichment around promoters for XPC-positively regulated genes (Fig. 5a). ChIP assays on *CCND1* and *DAPK1* promoters showed the recruitment of E2F1 after ATRA treatment only at the XPC-positively regulated genes in XP-C^WT^ cells (Fig. 5b and also S5B for additional genes). Moreover, the enrichment was lost in the absence of XPC in XP-C^DEL^ cells while it was still detected in XP-C^MUT^ cells (Fig. 5b and also Supplementary Figure 5B for additional genes).

We next assessed the existence of a complex containing XPC, KAT2A, and E2F1, by carrying out E2F1-immunoprecipitation experiments on nuclear extracts from untreated XP-C^WT^ cells. In these conditions, KAT2A and XPC co-precipitated a complex containing E2F1 (Fig. 5c). We thus investigated whether the detection of the complex, including XPC/hHR23B, KAT2A, and E2F1 reflected direct interactions between these proteins by using recombinant purified proteins (Fig. 5d). We observed that immunoprecipitation of KAT2A yielded in co-precipitated XPC alone as expected or E2F1 alone as previously showed^24^ (Fig. 5d, lanes 10 and 12) and that a complex containing recombinant and purified XPC/hHR23B, KAT2A, and E2F1 was detected when they were incubated together (Fig. 5d, lane 13).

Our data showed that the presence of responsive element for E2F1 defines the XPC-positively regulated genes and that XPC/hHR23B, E2F1, and KAT2A can interact to form a tripartite complex.

### XPC and E2F1 mediate the recruitment of KAT2A to XPC-regulated genes

**Fig. 6:**
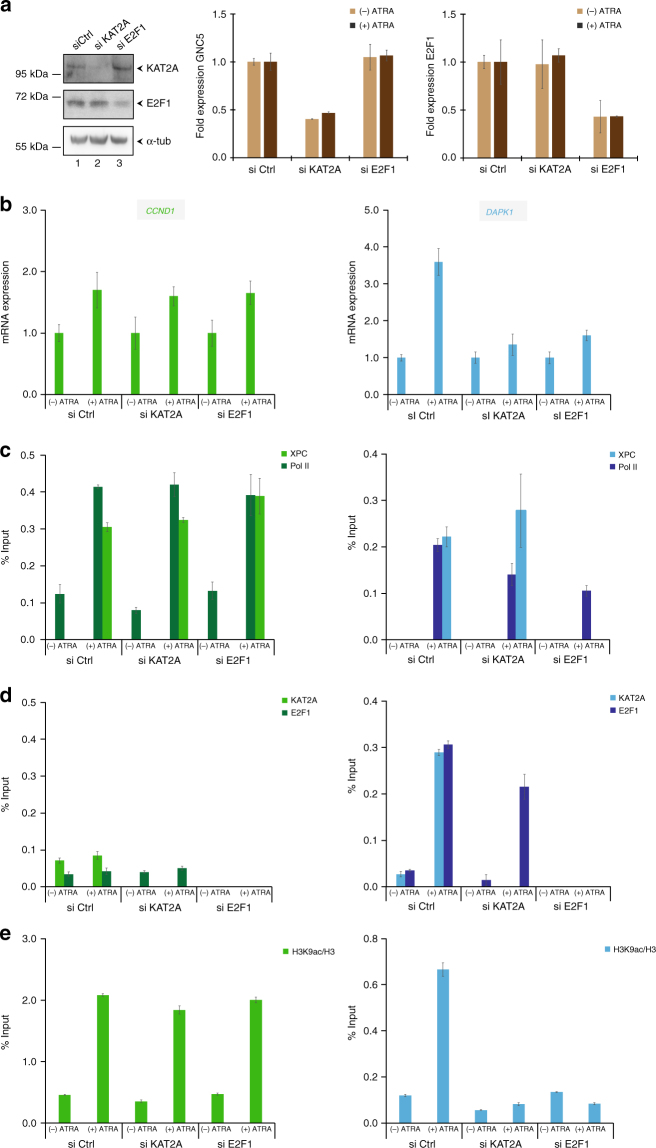
XPC and E2F1 are mutually necessary for their recruitment and co-operate to recruit KAT2A at promoters upon transcription. **a** Expression of KAT2A and E2F1 in XP-C^WT^ cells treated with siRNA targeting KAT2A (si KAT2A), E2F1 (si E2F1), or scrambled siRNA (si Ctrl) monitored by western blot (left panel) and qPCR (middle and left panels). **b** Relative mRNA expression of *CCND1* and *DAPK1*, before and after ATRA treatment in si Ctrl, si KAT2A, and si E2F1 XP-C^WT^ cells. **c**, **d**, **e** Corresponding recruitment of Pol II, XPC (**c**), KAT2A, and E2F1 (**d**) as well as histone H3K9ac/H3 (**e**) at *CCND1* and *DAPK1* promoters monitored by ChIP. All the error bars represent the standard deviation of three independent experiments

We next measured the impact of E2F1 and KAT2A on XPC-regulated gene expression and protein recruitment at promoters, by transiently transfecting XP-C^WT^ cells with siRNA targeting either KAT2A or E2F1 (Fig. 6a). Upon ATRA treatment, the silencing of KAT2A and E2F1 did not disturb the induction of *CCND1*, while *DAPK1* transactivation was abolished (Fig. 6b). ChIP analysis of the KAT2A and E2F1-independent *CCND1* promoter showed that their absence did not affect the recruitment of Pol II and XPC or the deposition of H3K9ac upon transcription (Fig. 6c, e, left panel). The silencing of KAT2A did not lead to significant changes in Pol II, XPC, and E2F1 recruitment at the *DAPK1* promoter (Fig. 6c, d, right panel). However, the recruitment of Pol II, XPC, and KAT2A decreased upon depletion of E2F1 (Fig. 6c, d, right panel). Both si KAT2A and si E2F1 blocked the acetylation of H3K9 at the *DAPK1* promoter (Fig. 6e). Similar results were obtained with *RARß2* (Supplementary Figure 6).

Together, these results showed that XPC, E2F1, and KAT2A are necessary for the appropriate acetylation of H3K9 at the promoter of XPC-positively regulated genes. Moreover, XPC and E2F1 were mutually necessary for their recruitment and drove KAT2A recruitment upon transactivation.

## Discussion

The present work uncovers the genomic distribution and the transcriptional impact of the DNA repair factor XPC. Since XPC is not involved in TC-NER^25^ and our study is performed in the absence of any exogenous genomic stress, the impact of XPC in modulating the transcriptional cell program is independent from its role in DNA repair. In the absence of damage and upon ATRA treatment the lesion sensor XPC was mainly localized around TSSs, collocating with Pol II and regulating positively or negatively around 500 genes. RNA-seq analysis also showed that several hundred genes were deregulated in XPC-depleted cells in the absence of ATRA treatment (Supplementary Figure 7A-B). The comparison between untreated and ATRA-treated conditions in both cells indicated a weak overlap between the different sets of deregulated genes (Supplementary Figure 7C and Supplementary Data 5). The expression of two gene models used in this study was unaffected by the absence of XPC in untreated cells, arguing that XPC only affects their transcription in the complex transcriptional context that represent RAR-dependent transactivation. In accordance to the GO analysis, our results describe phenomenon related to ATRA treatment but the data from untreated cells also showed that our observations are not exclusive to this stimulus. Further experiments will help to characterize the global relevance of the transcriptional role of XPC in transcription and its impact on the pathophysiology of XP-C patients.

For the purpose of clarity and accuracy, we deliberately focused our analysis on ATRA-dependent genes affected by the absence of XPC (representing 46% of all XPC peaks) although our preliminary data suggested the presence of XPC on other DNA regulatory regions such as enhancers, a phenomenon previously shown for pluripotency regulating genes in ES cells^15^.

The consequences of XPC depletion measured in fibroblasts derived from XP-C patients allowed us to identify, at the genomic scale, its importance for the enrichment of Pol II at TSSs and its specific involvement in the proper distribution of histone PTMs, including H3K9ac and H3K4me3 marks. Importantly, the analysis of shXPA cells indicated that the enrichment of the histone PTMs upon transcription was specifically related to the presence of XPC.

Using ex vivo and in vitro approaches, we demonstrated that XPC interacted with KAT2A and mediated its recruitment and the related H3K9ac deposition at promoter of XPC-positively regulated genes. The co-recruitment of XPC and E2F1 to promoter leads to the recruitment of KAT2A that is present in the SAGA and ATAC coactivator complexes. SAGA and ATAC also contain the close paralog of KAT2A, KAT2B, but only the KAT2A version of these complexes contains XPC. Furthermore, we observed that only ATAC was found to co-localize with XPC upon ATRA treatment so that the interaction of XPC with SAGA and its molecular role remains elusive and requires further investigation. We hypothesize that the SAGA-XPC assembly may target promoters regulated by other specific transcription factors and/or by different signaling cascades since ATAC and SAGA are recruited to different stress-induced genes promoters^23^.

Experiments performed in cells depleted in XPA show that XPC was still recruited to the pre-initiation complex (PIC) while depletion of XPC impaired the recruitment of XPA, indicating that XPC was the first NER factor to be recruited to the PIC. We next sought to identify the signals that triggered the recruitment of XPC to a subset of genes and identified the consensus binding site of E2F1 at 89% of XPC-activated genes. Using ChIP, we demonstrated that E2F1 and XPC mutually synergized their binding to promoters and consequently contribute both to the recruitment of KAT2A through direct interactions. XPC is then functioning as a bridge between E2F1 and ATAC, increasing the recruitment of the HAT to XPC-activated genes. As thus, XPC could be considered as a cofactor of mRNA expression in mammalian cells, linking a transcription factor (E2F1) to a coactivator (ATAC). This model also implies that all the mutations affecting XP-C patients are not affecting its transcription function, which is what we observed in XP-C^MUT^ cells that harbor a punctual mutation (XPC p.Pro334His) affecting XPC NER function without impact on RAR-dependent gene activation. In agreement with this observation, we didn’t observe impact of XPC p.Pro334His mutation on XPC/KAT2A interaction.

In addition to E2F1, we cannot exclude the role of additional players for the proper recruitment of XPC to TSS, similarly to TATA-binding protein associated factors (TAFs) that were shown to interact with XPF^26,27^. Interestingly, among the genes positively regulated by XPC, *SET1* encoding one methyl transferase depositing H3K4me3 mark has been identified. Concomitantly, a reduced expression of SET1 and a reduced recruitment of the protein at genes activated by XPC were observed in XP-C^DEL^ cells, suggesting an indirect effect of XPC on the H3K4me3 mark (Supplementary Figure 8).

Based on our genome-wide analysis, a large proportion of genes regulated by XPC are related to chromatin and present oncogenic and immunologic signatures. XPC seems to be related to the immune response as demonstrated in two recent studies. First, it has been demonstrated that XPC, through DNA damage, can induce the expression of cytokine like interleukin-6 possessing pro-tumorigenic effects in lung fibroblasts^28^. Also, multi-omic analyses aiming to identify factors and pathways implicated in the cellular response to UV-induced DNA damage have recently shown connections with the immune system^29^. In this study, the analysis of several IFN mRNA expression in XP-C^WT^ and XP-C^DEL^ cells in absence of ATRA treatment indicated higher expressions of IFN beta, gamma, and alpha in cells XPC-depleted that were unrelated to the RAR-dependent pathway (Supplementary Figure 9). Although the dual molecular involvement of NER factors in transcription and DNA repair is now well established, the impact of their deregulation in the pathological context is still elusive, including for the immune response of XP patients. What is more, our data suggest an additional link with transcriptional deficiencies. *DAPK1*, analyzed in this study, is particularly interesting as it acts as an inhibitor of RIG-I, signaling the necessity to induce the production of type I IFN^30^ and as a tumor suppressor, downregulated in multiple cancer types^31^.

Our comparison of fibroblasts derived from two XP-C patients XP-C^DEL^ and XP-C^MUT^, bearing XPC p.Arg579* and XPC p.Pro334His mutations, respectively, presenting severe and mild clinical features, respectively, shows deregulation of gene expression only in XP-C^DEL^ cells while GG-NER is impaired in the two cell lines^17,18^. In XP-C^DEL^ cells indeed, the absence of XPC impairs the DNA damage recognition step while in XP-C^MUT^ cells, the XPC variant can still bind the damaged sites, recruits TFIIH but impedes the stimulation of the TFIIH XPB subunit ATPase activity, leading to a delayed arrival of XPA^17^. Our observations suggest a correlation between transcriptional default and XP severity although more XP-C cases need to be tested to support this hypothesis. However, the pathological implication of the deregulation of the XPC transcriptional roles remains questioning. Indeed, although XPC has been identified in a transcriptional coactivator complex containing Oct4/Sox2 that maintains pluripotency of stem cells^15^, it has to be noticed that XPC^−/−^ mice do not show developmental defects^32^. Also, ablation of the C-terminal region of XPC gene abrogating the interaction sites of XPC with known partners RAD23 and CETN2 has minimal impact on gene expression or pluripotency to contribute to chimeric embryos^33^. The identification of genes and the pathways regulated by XPC will give a more global view of the role of XPC as a Pol II cofactor and will help to identify relevant markers for an early and specific diagnosis, and to anticipate/predict the cancer risk among the different symptoms. In conclusion, we have uncovered a role for XPC as a regulator of chromatin marks necessary for transcription, redefining the molecular etiology of XP.

## Methods

### Cell culture

HeLa Silencix cells (Tebu-Bio), including shCtrl, shXPC, and shXPA cells, MRC-5 fibroblasts (ATCC # CCL-171), XP-C patient-derived fibroblasts GM14867 (XP-C^DEL^) and GM02096 (XP-C^MUT^), and rescued XP-C (XP-C^WT^)^17,19^ (Coriell Institute) were used and cultured in appropriate medium. Twelve hours before ligand treatment, cells were incubated with phenol red-free medium containing charcoal-treated fetal calf serum and 40 mg/ml gentamycin. Cells were treated with 10 μM ATRA (MP).

### Reverse transcription and quantitative PCR

Total RNA was isolated from several cell lines using a GenElute Mammalian Total RNA Miniprep kit (Sigma) and reverse transcribed with SuperScript IV reverse transcriptase (Invitrogen). The quantitative PCR (qPCR) was done using the Lightcycler 480 (Roche). The primer sequences for the different genes used in qPCR are indicated in Supplementary Data 3. The mRNA expression of the different analyzed genes represents the ratio between values obtained from treated and untreated cells normalized with the housekeeping *GAPDH* mRNA.

### Chromatin immunoprecipitation

Cells were crosslinked at room temperature for 10 min with 1% formaldehyde. Chromatin was prepared and sonicated at 4 °C for 30 min using sonicator Q800R (Qsonica) as previously described^6^. Samples were immunoprecipitated with antibodies at 4 °C overnight, and protein G-coated Dynabeads (Invitrogen) were added, incubated for 4 h at 4 °C, and sequentially washed. Protein-DNA complexes were eluted and decrosslinked. DNA fragments were purified using QIAquick PCR purification kit (QIAGEN) and analyzed by qPCR using a set of primers indicated in Supplementary Data 3.

### RNA-seq analysis

Total RNA from XP-C^WT^ and XP-C^DEL^ cells were extracted before or 6 h after ATRA treatment (10 μM) using TRI REAGENT (MRC) and purified by phenol-chloroform extraction. Libraries were prepared with TruSeq Stranded mRNA Sample Preparation kit following guide instruction and subsequently proceed on an Illumina Hiseq 4000 as single-end 50 base reads following Illumina’s instructions. Image analysis and base calling were performed using RTA 2.7.3 and bcl2fastq 2.17.1.14. Reads were mapped onto the hg19 assembly of the human genome. Reads count was performed with HOMER v4.8.3(65) and expression was estimated with EdgeR. Genome ontology was performed with The Database for Annotation and Integrated Discovery v6.7 (https://david.ncifcrf.gov).

### ChIP-seq analysis

Purified DNA fragments analyzed by ChIP-seq were prepared by using the ChIP-IT High Sensitivity Kit (Active Motif) and the related antibodies. ChIP-seq was performed on an Illumina Hiseq 2500 as single-end 50 base reads following Illumina’s instructions. Image analysis and base calling were performed using RTA 1.17.20 and CASAVA 1.8.2. Reads were mapped onto the hg19 assembly of the human genome. Peak detection was performed using MACS (http://liulab.dfci.harvard.edu/MACS/) under settings where the input fraction was used as negative control. Peaks detected were annotated using HOMER (http://biowhat.ucsd.edu/homer/ngs/annotation.html) as well as TSS protein enrichment comparison. Quantitative comparison of Pol II gene body enrichment was performed using seqMINER (http://bips.u-strasbg.fr/seqminer/). As reference coordinates, we used the MACS-determined peaks or the annotated TSS/TTS of human genes as defined by RefSeq database. Sequence enrichment was performed using RSAT (http://rsat.sb-roscoff.fr) with MACS-determined peaks as reference.

### Plasmids and purification of recombinant proteins

PCR products for the entire coding sequence XPC was cloned into pDONOR-207 vector using the Gateway system (Invitrogen) and later cloned in bicistronic plasmid VEAP5317 with hHR23B kindly obtained from A. Poterszman. For recombinant Flag-GCN5 expression in Sf9 cells, the corresponding vector pSK227-KAT2A was kindly provided by L. Tora^34^. PCR product for the entire coding sequence E2F1 was also cloned into pDONOR-207 vector and later sub-cloned in pAC8 vector. Sf9 cells were infected with baculoviruses expressing a FLAG-tagged KAT2A, Strep tactin-tagged XPC/His-tagged hHR23B, or c-myc-tagged E2F1, and the harvested recombinant proteins were purified as previously described^35^.

### siRNA transfection

ON-TARGET plus smart pool siRNA control or targeting human KAT2A and E2F1 were purchased from Thermo Scientific and transfected in XP-C^WT^ cells at a final concentration of 100 nM using X-tremeGENE siRNA transfection reagent (Sigma-Aldrich) following the manufacturer’s protocol.

### Co-immunoprecipation

For in vivo co-IPs, nuclear extracts from untreated XP-C^WT^ and XP-C^DEL^ cells were prepared as previously described^36^. After GFP-trap or KAT2A, ZZZ3, or TRRAP immunoprecipitation using the appropriate antibodies conjugated to protein G-coated Dynabeads (Invitrogen), followed by extensive washes, was carried out and the different co-precipitated proteins were detected using specific antibodies after immunoblotting.

For in vitro co-IPs, recombinant purified Flag-KAT2A was then incubated with recombinant purified Strep-XPC/His-hHR23B and/or c-myc E2F1 before Flag immunoprecipitation was carried out. After washes, bound proteins were resolved by SDS–polyacrylamide gel electrophoresis and detected by western blot.

### Antibodies

Antibodies toward RBP1 (RNA Pol II) (7C2), RAR (9A6), TBP (3G3), XPA (1E11), KAT2A (5GC-2A6), XPG (1B5), ZZZ3 (2616), XPC (2076), and TRRAP (1B3) were produced at the IGBMC. CDK7 (C-19), E2F1 (C-20), and XPC (D-18) antibodies were purchased by Santa-Cruz Biotechnology. H3k4me3 (ab1012), KAT2B (ab12188), and α-tubulin (ab15246) antibodies were purchased by Abcam and WDR5 (07–706) antibody from Upstate. Antibodies directed toward KAT2A (A4013) were obtained from Epigentek while those against Spt7L (A302–803A) and hSET1 (A300–803A) from Bethyl. Antibodies against H3K9ac (61251) and H3 (61475) were from Active Motif. Antibodies toward GFP (TP401) and M2-Flag (F1804) were purchased by Torrey Pines Biolabs and Sigma-Aldrich, respectively. For immunoblot, the concerned antibodies were diluted at 1–1000 while 5–10 μg of antibodies were used for ChIP experiments.

### Data availability

All relevant data are available from the authors upon request. The accession numbers for the sequencing data reported in this paper are https://www.ncbi.nlm.nih.gov/Traces/study/?acc=SRP148864.

## Electronic supplementary material

Supplementary Information

Description of Additional Supplementary Files

Supplementary Data 1

Supplementary Data 2

Supplementary Data 3

Supplementary Data 4

Supplementary Data 5
