# Supplementary material for: XPC is an RNA polymerase II cofactor recruiting ATAC to promoters by interacting with E2F1
Source: Nat Commun. 2018 Jul 4;9:2610. doi: 10.1038/s41467-018-05010-0 (PMC6031651; doi:10.1038/s41467-018-05010-0)
Supplement: Supplementary file 1 — Supplementary Information [file 41467_2018_5010_MOESM1_ESM.pdf]

**Supplementary Methods**

**Microarray analysis**

Total RNA from shXPC, XP-CDEL and shCtrl cells were extracted before or 6 hours after t-RA treatment (10µM) using GenElute Mammalian Total RNA Miniprep kit (Sigma). Two independent experiments were performed. RNA quality was verified by analysis on the 2100Bioanalyzer (Agilent). Biotinylated single strand cDNA targets were prepared, starting from250 ng of total RNA, using the Ambion WT Expression Kit (Cat # 4411974) and the Affymetrix GeneChip® WT Terminal Labeling Kit (Cat # 900671), according to Affymetrix recommendations. Following fragmentation and end-labeling, 1.9 µg of cDNAs were hybridized for 16 hours at 45°C on GeneChip® Human Gene 1.0 ST arrays (Affymetrix) interrogating 28,853 genes represented by approximately 27 probes spread across the full length of the gene. The chips were washed and stained in the GeneChip® Fluidics Station 450 (Affymetrix) and scanned with the GeneChip® Scanner 3000 7G (Affymetrix). Finally, raw data (CEL Intensity files) were extracted from the scanned images using the Affymetrix GeneChip® Command Console (AGCC) version 3.1. CEL files were further processed with Affymetrix Expression Console software version 1.1 to calculate probeset signal intensities using Robust Multi-array Average (RMA) algorithms with default settings.

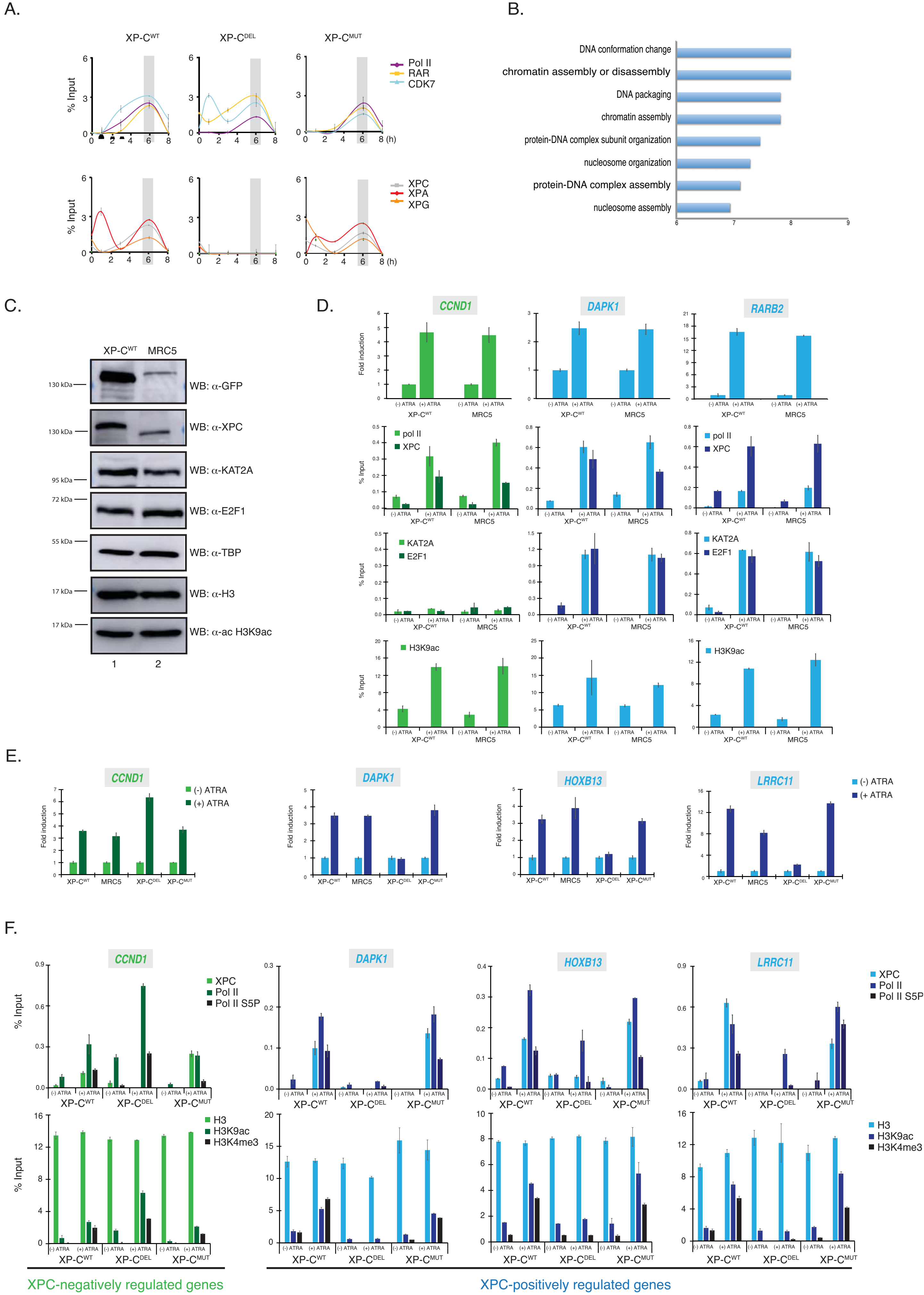

A.
XP-C^WT
XP-C^DEL
XP-C^MUT
% Input
Pol II
RAR
CDK7
XPC
XPA
XPG
B.
DNA conformation change
chromatin assembly or disassembly
DNA packaging
chromatin assembly
protein-DNA complex subunit organization
nucleosome organization
protein-DNA complex assembly
nucleosome assembly
C.
XP-C^WT
MRC5
WB: α-GFP
WB: α-XPC
WB: α-KAT2A
WB: α-E2F1
WB: α-TBP
WB: α-H3
WB: α-ac H3K9ac
D.
CCND1
DAPK1
RARB2
Fold induction
pol II
XPC
KAT2A
E2F1
H3K9ac
E.
CCND1
DAPK1
HOXB13
LRRC11
(-) ATRA
(+) ATRA
F.
XPC
Pol II
Pol II S5P
H3
H3K9ac
H3K4me3
XPC-negatively regulated genes
XPC-positively regulated genes

**Supplementary Figure 1**: **NER factors, transcriptional machinery and histone PTMs at XPC target-genes and their gene-ontology annotations.**

**A-**ChIP experiments monitoring the occupancy of Pol II, RAR, CDK7, XPC, XPA and XPG at *RARβ2* promoter for up to 8 hours in chromatin extracts from XP-$C^{WT}$, XP-$C^{DEL}$ and XP-$C^{MUT}$ cells. Error Bars represent the standard deviation of three independent experiments.

**B-**Gene ontology annotation performed with DAVID 6.7 on XPC-positively regulated genes.

**C-** Relative protein expression of XPC, GFP-XPC, KAT2A, E2F1, TBP, H3 and H3K9ac analysed by Western Blot from whole cell and nuclear extracts from XP-$C^{WT}$ and MRC5 cells.

**D-** Fold induction of *RARβ2*, *DAPK1* and *CCND1* in XP-$C^{WT}$ and MRC5 fibroblasts, after ATRA treatment during 6 hours (upper panels). Occupancy of XPC, Pol II, KAT2A, E2F1 (central panels) and H3K9ac (lower panels) at promoters of XPC- negatively regulated gene *CCND1* and XPC-positively regulated genes *DAPK1* and *RARβ2*, monitored by ChIP from chromatin extracts of XP-$C^{WT}$ and MRC-5 cells in presence or absence of ATRA. All the Error Bars represent the standard deviation of three independent experiments.

**E-** Fold induction of XPC-positively regulated *DAPK1, HOXB13, LRCC11* and XPC-negatively regulated *CCND1* in XP-$C^{WT}$, XP-$C^{DEL}$ and XP-$C^{MUT}$ and MRC5 fibroblasts, after ATRA treatment during 6 hours. Error Bars represent the standard deviation of three independent experiments.

**F-** Occupancy of XPC, Pol II and Serine 5 phosphorylated Pol II (Pol II S5P) (Upper panel) or H3, H3K9ac and H3K4me3 (lower panel) at promoters of XPC-negatively regulated gene *CCND1* and XPC- positively regulated genes *DAPK1*, *HOXB13* and *LRRC11*, monitored by ChIP from chromatin extracts of XP-$C^{WT}$, XP-$C^{DEL}$ and XP-$C^{MUT}$ cells in presence or absence of ATRA. Error Bars represent the standard deviation of three independent experiments.

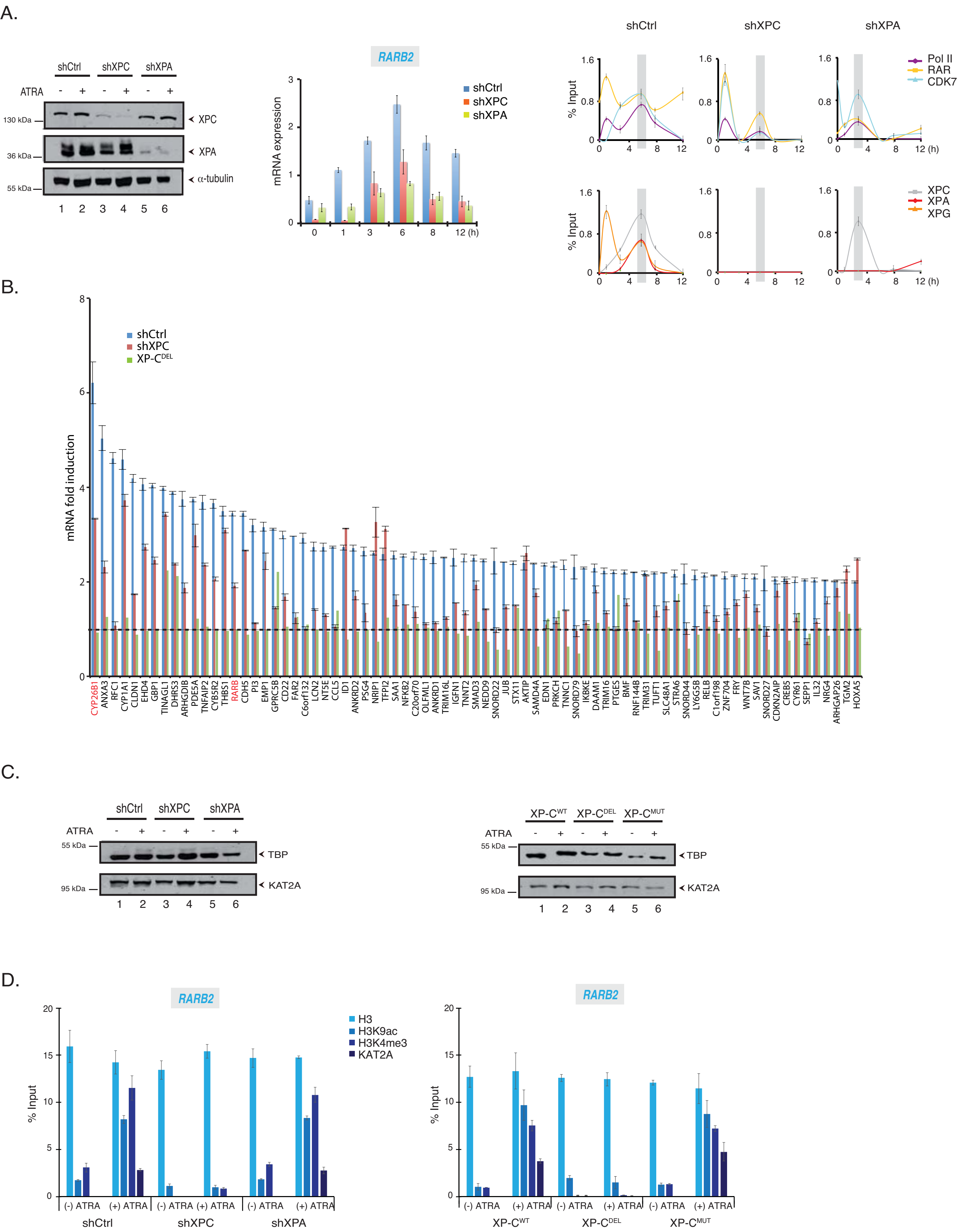
A.
shCtrl
shXPC
shXPA
ATRA
130 kDa
36 kDa
55 kDa
XPC
XPA
α-tubulin
1 2 3 4 5 6
RARB2
mRNA expression
0 1 3 6 8 12 (h)
% Input
Pol II
RAR
CDK7
XPC
XPA
XPG
0 4 8 12 (h)
B.
mRNA fold induction
shCtrl
shXPC
XP-C^DEL
CYP26B1 ANXA3 RFC1 CYP1A1 CLDN1 EHD4 GBP1 TINAGL1 DHRS3 ARHGDIB PDE5A TNFAIP2 CYB5R2 THBS1 RARB CDH5 PI3 EMP1 GPRC5B CD22 FAR2 C6orf132 LCN2 NT5E CCL5 ID1 ANKRD2 PSG4 NRIP1 TFPI2 SAA1 NFKB2 C20orf70 OLFML1 ANKRD1 TRIM16L IGFN1 TNNT2 SMAD3 NEDD9 SNORD22 JUB STX11 AKTIP SAMD4A EDN1 PRKCH TNNC1 SNORD79 IKBKE DAAM1 TRIM16 PTGES BMF RNF144B TRIM31 TUFT1 SLC48A1 STRA6 SNORD44 LY6G5B RELB C1orf198 ZNF704 FRY WNT7B SAV1 SNORD27 CDKN2AIP CREB5 CYR61 SEPP1 IL32 NRG4 ARHGAP26 TGM2 HOXA5
C.
shCtrl
shXPC
shXPA
ATRA
55 kDa
95 kDa
TBP
KAT2A
1 2 3 4 5 6
XP-C^WT
XP-C^DEL
XP-C^MUT
D.
RARB2
% Input
H3
H3K9ac
H3K4me3
KAT2A
(-) ATRA
(+) ATRA
shCtrl
shXPC
shXPA
XP-C^WT
XP-C^DEL
XP-C^MUT

**Supplementary Figure 2**: **Characterization of active Histone PTMs marks H3K9ac and H3K4me3 in XP-C$^{WT}$, XP-C$^{DEL}$ and XP-C$^{MUT}$ cells and characterization of shCtrl, shXPC and shXPA cells for NER factors and transcriptional machinery.**

**A-** Relative protein expression of XPC and XPA analysed by Western Blot from whole cell extract from shCtrl, shXPC or shXPA HeLa cells, in presence or absence of ATRA (left panel). Relative mRNA expression of *RARβ2* in shCtrl, shXPC or shXPA HeLa cells after ATRA treatment in 12 hours' time course experiment (central panel). Corresponding recruitment of Pol II, RAR, CDK7, XPC, XPA and XPG at *RARβ2* promoter for up to 12 hours as monitored by ChIP (right panel). Error bars represent the standard deviation of three independent experiments.

**B-** Transcriptome profiling of shCtrl (blue), shXPC (red) and XP-C$^{DEL}$ (green) cells treated with ATRA during 6 hours using the whole transcript coverage Affymetrix Human Gene 1.0 ST arrays. Based on two independent experiments, these genes show an up-regulation > to 2 (±SEM) compared to t=0 hour (F-test, $P<0.005$).

**C-** Protein expression of KAT2A and TBP analysed by Western Blot from whole cell extracts from shCtrl, shXPC or shXPA HeLa cells (left panel) and from XP-C$^{WT}$, XP-C$^{DEL}$ and XP-C$^{MUT}$ fibroblasts (right panel), in presence or absence of ATRA.

**D-** ChIP experiment monitoring for recruitment of KAT2A and deposition of Histone H3, H3K9ac, H3K4me3 at *RARβ2* promoter using chromatin extracts from shCtrl, shXPC or shXPA HeLa cells (left panel) and XP-C$^{WT}$, XP-C$^{DEL}$ and XP-C$^{MUT}$ fibroblasts (right panel), in presence or absence of ATRA. Error bars represent the standard deviation of three independent experiments.

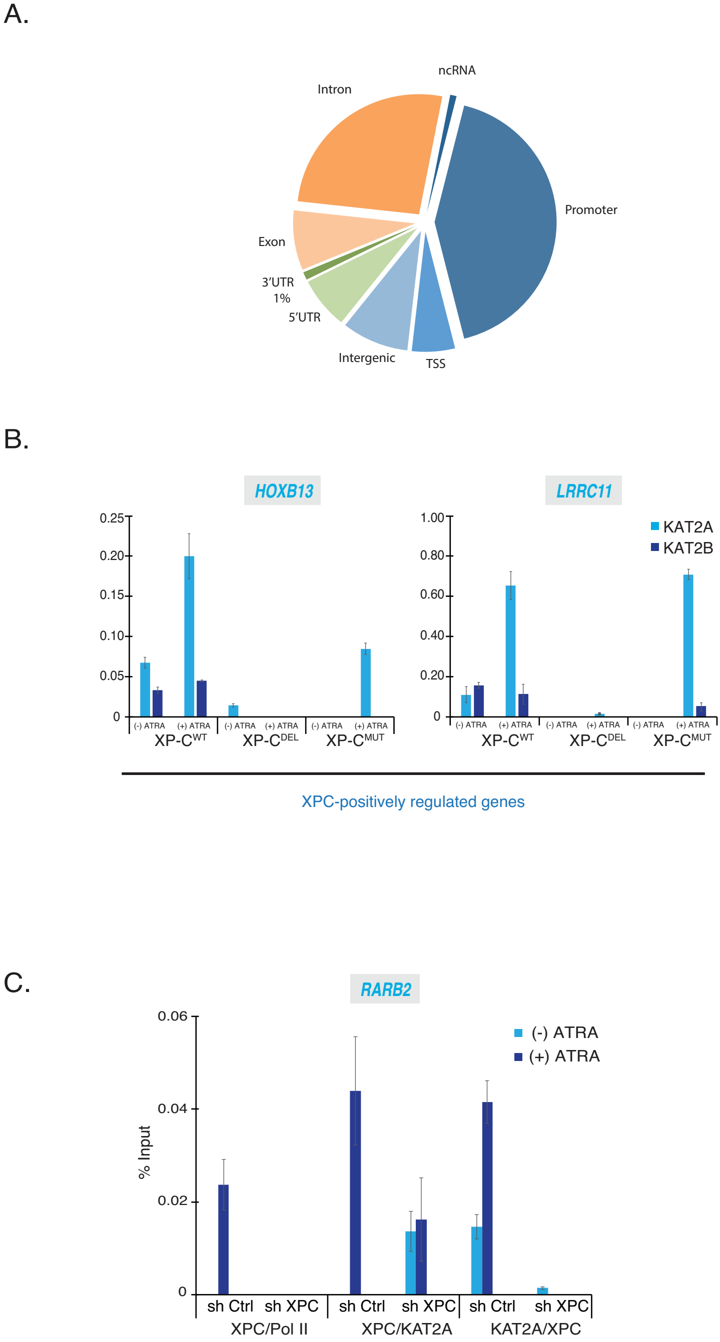

A.
ncRNA
Intron
Promoter
Exon
3'UTR
1%
5'UTR
Intergenic
TSS
B.
HOXB13
LRRC11
KAT2A
KAT2B
0.25
0.20
0.15
0.10
0.05
0
1.00
0.80
0.60
0.40
0.20
0
(-) ATRA
(+) ATRA
XP-C^WT
XP-C^DEL
XP-C^MUT
XPC-positively regulated genes
C.
RARB2
(-) ATRA
(+) ATRA
0.06
0.04
0.02
0
% Input
sh Ctrl
sh XPC
XPC/Pol II
XPC/KAT2A
KAT2A/XPC

**Supplementary Figure 3: KAT2A is recruited XPC-positively regulated genes**

**A-** HOMER annotation of KAT2A.

**B-** ChIP experiments monitoring the occupancy of KAT2A and KAT2B at promoters of XPC-positively regulated genes *HOXB13* and *LRRC11* from chromatin extracts of XP-C$^{WT}$, XP-C$^{DEL}$ and XP-C$^{MUT}$ cells in presence or absence of ATRA. Error bars represent the standard deviation of three independent experiments.

**C-** ChIP/Re-ChIP experiment monitoring the co-occupancy of either XPC and Pol II or XPC and KAT2A in shCtrl and shXPC HeLa cells at *RARβ2* promoter upon ATRA treatment. Error bars represent the standard deviation of three independent experiments.

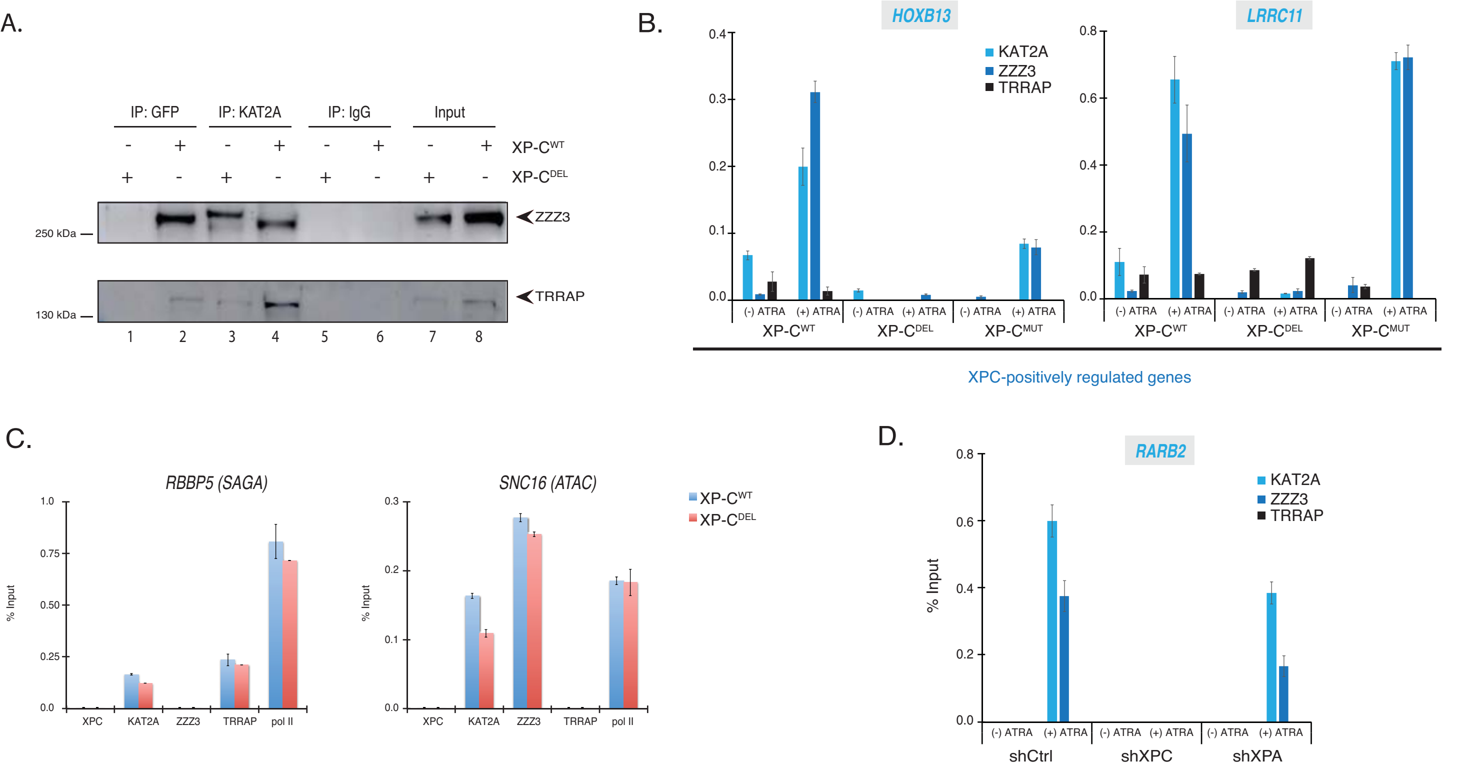
A.
IP: GFP
IP: KAT2A
IP: IgG
Input
XP-CWT
XP-CDEL
ZZZ3
TRRAP
250 kDa
130 kDa
1 2 3 4 5 6 7 8
B.
HOXB13
LRRC11
KAT2A
ZZZ3
TRRAP
(-) ATRA
(+) ATRA
XP-CWT
XP-CDEL
XP-CMUT
XPC-positively regulated genes
C.
RBBP5 (SAGA)
SNC16 (ATAC)
% Input
XPC
KAT2A
ZZZ3
TRRAP
pol II
XP-CWT
XP-CDEL
D.
RARB2
% Input
KAT2A
ZZZ3
TRRAP
(-) ATRA
(+) ATRA
shCtrl
shXPC
shXPA

**Supplementary Figure 4**: **XPC is found in both ATAC and SAGA complexes but only ATAC was detected at XPC-target promoters upon transcription.**

**A-** Immunoprecipitation performed on nuclear extracts from XP-$C^{WT}$ or XP-$C^{DEL}$ fibroblasts with antibody against GFP, KAT2A or IgG. Western Blot was revealed with antibodies directed against ZZZ3 (ATAC) and TRRAP (SAGA).

**B-** ChIP experiment investigating the occupancy of KAT2A, ZZZ3 and TRRAP at promoters of XPC-positively regulated genes *HOXB13* and *LRRC11* from chromatin extracts of XP-$C^{WT}$, XP-$C^{DEL}$ and XP-$C^{MUT}$ cells in presence or absence of ATRA. Error bars represent the standard deviation of three independent experiments.

**C-** ChIP experiment monitoring the occupancy of XPC, KAT2A, ZZZ3, TRRAP and Pol II at *RBBP5* (targeted by SAGA) and *SNC16* (targeted by ATAC) promoters using chromatin extracts from XP-$C^{WT}$ and XP-$C^{DEL}$ fibroblasts. Error bars represent the standard deviation of three independent experiments.

**D-** ChIP experiment analysing the recruitment of KAT2A, ZZZ3 and TRRAP at *RARβ2* promoter using chromatin extracts from shCtrl, shXPC or shXPA HeLa cells, before and after ATRA treatment. Error bars represent the standard deviation of three independent experiments.

A.

| | Raw P-Value | Hyper FDR Q-Val | Total Genes |
|---|---|---|---|
| Motif TTTCSCGC matches E2F1: E2F transcription factor 1 | 1.991889e-12 | 3.300984825e-9 | 254/283 |

B.

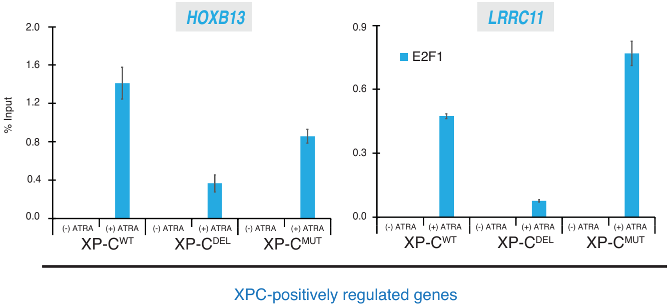

**Supplementary Figure 5: XPC-positively regulated genes harbour an E2F1 signature.**

**A-** Motif research analysis from XPC negatively-regulated and positively-regulated genes promoter sequences using GREAT.

**B-** ChIP experiment investigating the occupancy of E2F1 at promoters of XPC-positively regulated genes *HOXB13* and *LRRC11* from chromatin extracts of XP-$C^{WT}$, XP-$C^{DEL}$ and XP-$C^{MUT}$ cells in presence or absence of ATRA. Error bars represent the standard deviation of three independent experiments.

A.

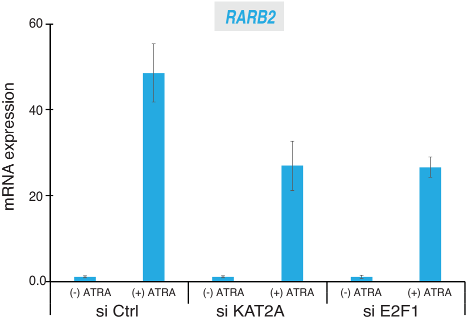

RARB2
mRNA expression
60
40
20
0.0
(-) ATRA
(+) ATRA
si Ctrl
si KAT2A
si E2F1


B.

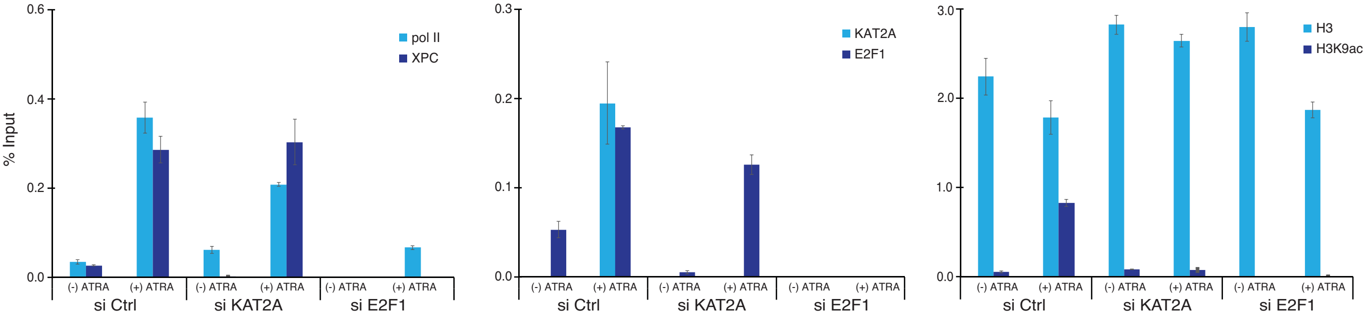

% Input
0.6
0.4
0.2
0.0
pol II
XPC
0.3
0.2
0.1
KAT2A
E2F1
3.0
2.0
1.0
H3
H3K9ac
(-) ATRA
(+) ATRA
si Ctrl
si KAT2A
si E2F1

**Supplementary Figure 6**: **XPC and E2F1 are mutually necessary for their recruitment and co-operate to recruit KAT2A at *RAR*b*2* promoter upon transcription.**

**A-** Relative mRNA expression of *RARβ2* before and after ATRA treatment in si Ctrl, si KAT2A and si E2F1 XP-$C^{WT}$ cells. Error bars represent the standard deviation of three independent experiments.

**B-** Corresponding recruitment of Pol II, XPC, KAT2A and E2F1 as well as histone H3 and H3K9ac at *RARβ2* promoter monitored by ChIP. Error bars represent the standard deviation of three independent experiments.

**A.**

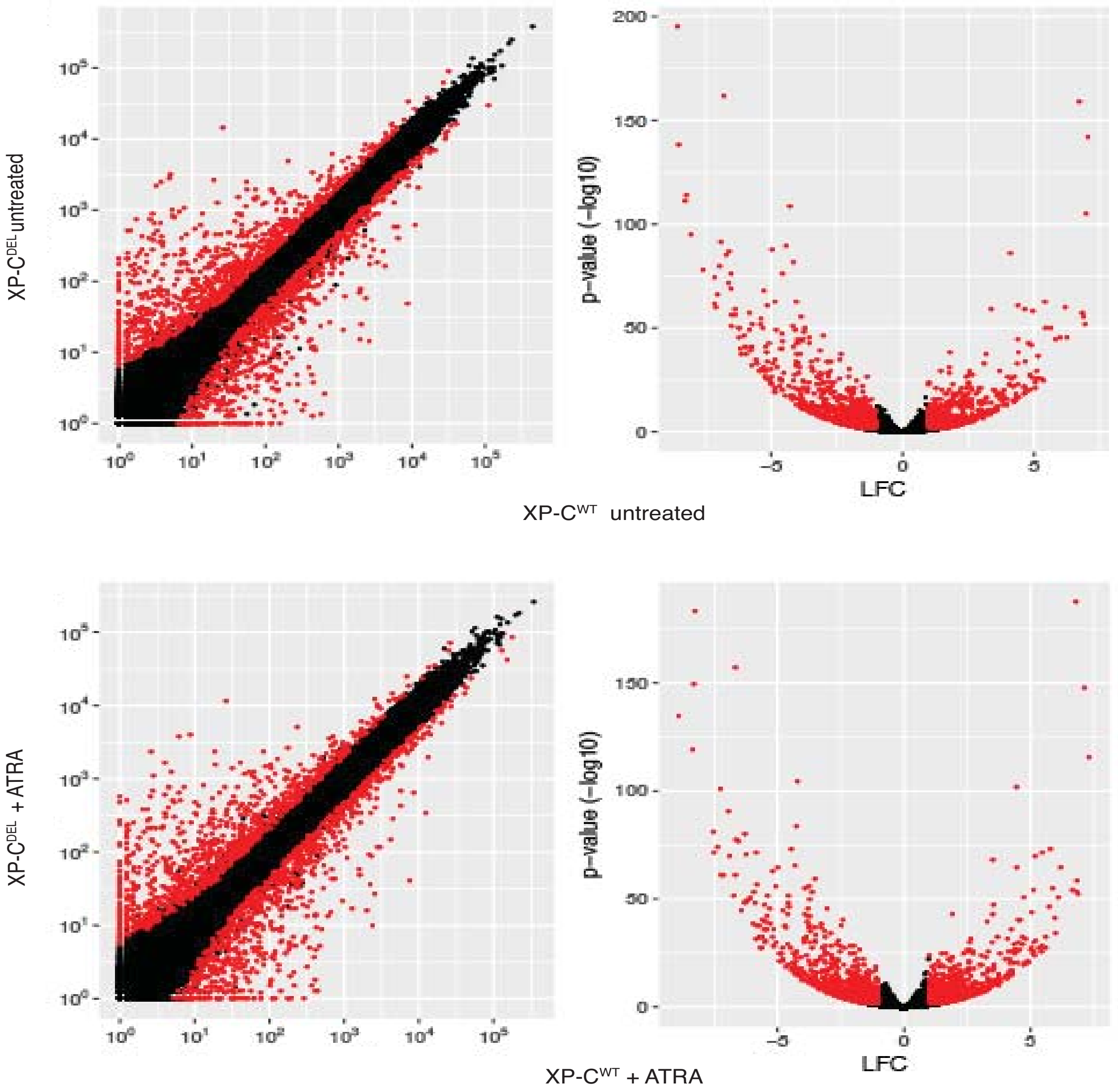


**B.**

| | Over-expressed | Under-expressed | Total |
|---|---|---|---|
| XP-C$^{WT}$ vs XP-C$^{DEL}$ untreated | 824 | 944 | 1,768 |
| XP-C$^{WT}$ vs XP-C$^{DEL}$ +ATRA | 868 | 985 | 1,853 |

**C.**

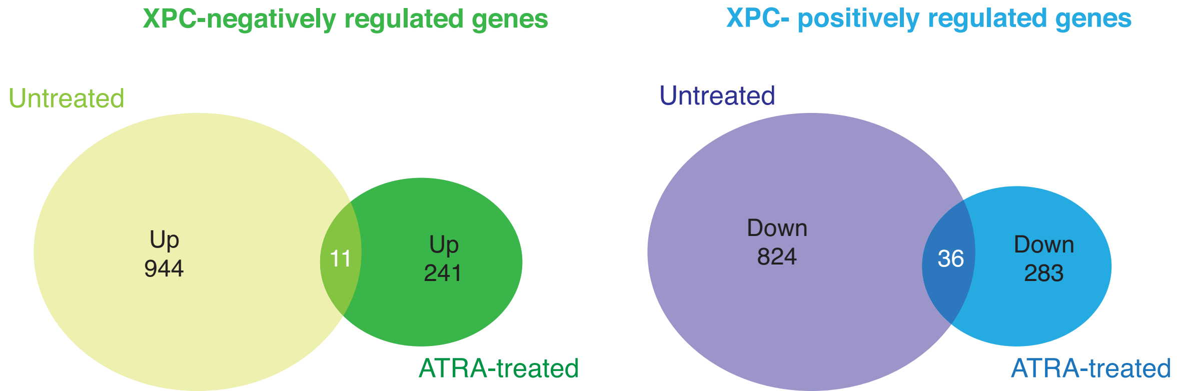

**Supplementary Figure 7: XPC regulates positively and negatively subsets of genes in absence or presence of ATRA treatment.**

**A-**Comparison of XP-C$^{WT}$ and XP-C$^{DEL}$ with or without ATRA by RNA-seq. Red points show significant data. All data have been evaluated with DESeq2 R package. Left upper panels and lower panels represent the normalized gene expression without ATRA or after ATRA treatment respectively. For a given gene, its value is obtained by mean on all samples belonging to the same condition plus one. Right upper and lower panels represent the volcano plot for cells without ATRA or after ATRA treatment respectively. The x-axis represents the LFC values and the y-axis the p-values.

**B**-Table summarizing the comparative data obtained from the RNA-seq described above between untreated and ATRA-treated XP-C$^{WT}$ and XP-C$^{DEL}$ fibroblasts indicating the numbers of over-expressed and under-expressed genes.

**C-**Venn Diagram indicating the number of genes commonly either negatively (green) or positively (blue) regulated by XPC in both untreated and ATRA-treated conditions.

A.

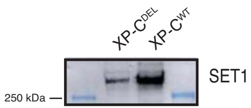

XP-C^DEL
XP-C^WT
SET1
250 kDa


B.

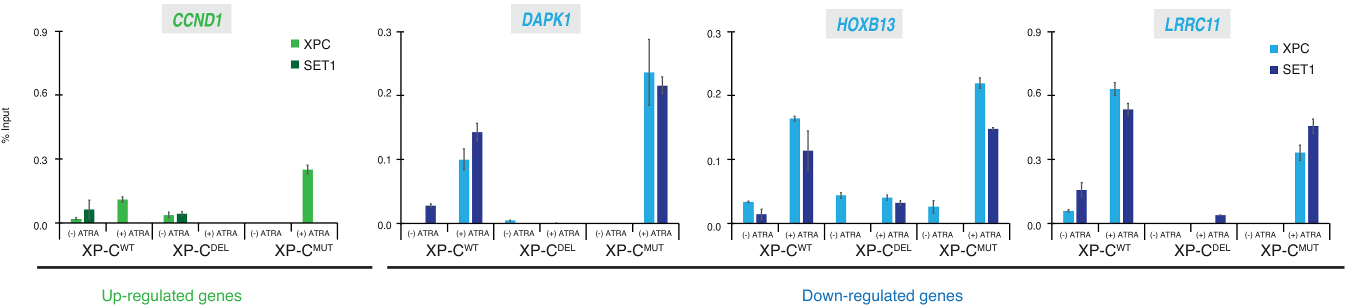

CCND1
DAPK1
HOXB13
LRRC11
XPC
SET1
% Input
0.9
0.6
0.3
0.0
0.2
0.1
(-) ATRA
(+) ATRA
XP-C^WT
XP-C^DEL
XP-C^MUT
Up-regulated genes
Down-regulated genes

**Supplementary Figure 8: XPC directly regulates the expression of SET1 and consequently the deposition of H3K4me3 at XPC-positively regulated promoters.**

**A-** Protein expression of SET1 and TBP analysed by Western Blot from whole cell extract from XP-$C^{WT}$ and XP-$C^{DEL}$ fibroblasts in presence or absence of ATRA.

**B-** ChIP experiment looking for occupancy of XPC and SET1 at *CCND1*, *DAPK1*, *HOXB13* and *LRRC11* promoters using chromatin extracts from XP-$C^{WT}$, XP-$C^{DEL}$ and XP-$C^{MUT}$ fibroblasts. Error bars represent the standard deviation of three independent experiments.

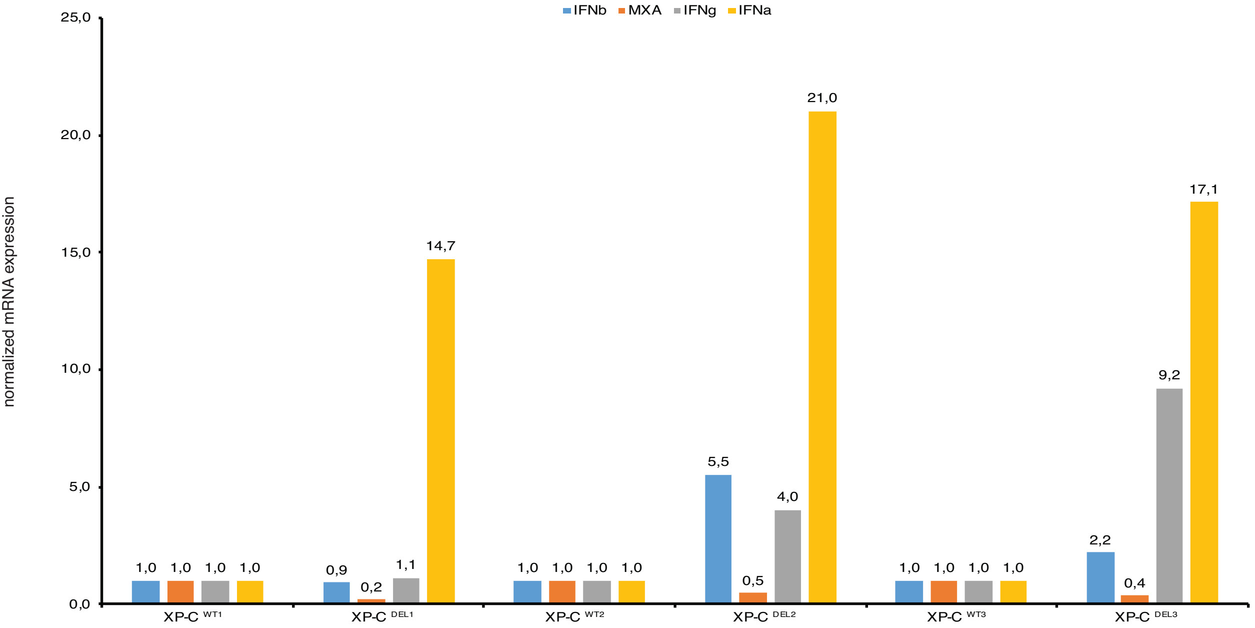
IFNb
MXA
IFNg
IFNa
normalized mRNA expression
25,0
20,0
15,0
10,0
5,0
0,0
1,0
1,0
1,0
1,0
0,9
0,2
1,1
14,7
1,0
1,0
1,0
1,0
5,5
0,5
4,0
21,0
1,0
1,0
1,0
1,0
2,2
0,4
9,2
17,1
XP-C WT1
XP-C DEL1
XP-C WT2
XP-C DEL2
XP-C WT3
XP-C DEL3

**Supplementary Figure 9: Untreated Human fibroblasts depleted with XPC highly express IFN compared to XP-C$^{WT}$.**

Relative mRNA expression of *IFN gamma* (*IFNg*)*, IFN beta (IFNb), IFN alpha* (*IFNa*) and *MXA* measured by qRT-PCR in untreated XP-C$^{WT}$ and XP-C$^{DEL}$ fibroblasts.
