# Supplementary material for: XPC is an RNA polymerase II cofactor recruiting ATAC to promoters by interacting with E2F1
Source: Nat Commun. 2018 Jul 4;9:2610. doi: 10.1038/s41467-018-05010-0 (PMC6031651; doi:10.1038/s41467-018-05010-0)
Supplement: Supplementary file 2 — Description of Additional Supplementary Files [file 41467_2018_5010_MOESM2_ESM.docx]

**Description of Additional Supplementary Files**

File Name: Supplementary Data 1

Description: List of XPC-regulated genes

File Name: Supplementary Data 2

Description: Gene-ontology annotation by GREAT of XPC-positively regulated genes

File Name: Supplementary Data 3

Description: List of primers and their sequences.

File Name: Supplementary Data 4

Description: List of genes downregulated upon ATRA treatment in shXPC and XPCDEL cells compared to shCtrl cells analysed by microarray.

File Name: Supplementary Data 5

Description: List of genes deregulated in untreated and ATRA-treated XP-CDEL cells analysed by RNA-seq.
